# The Effects of Heat Stress on the Ovary, Follicles and Oocytes: A Systematic Review

**DOI:** 10.1101/2024.12.04.626831

**Published:** 2024-12-05

**Authors:** Luhan T. Zhou, Dilan Gokyer, Krystal Madkins, Molly Beestrum, Daniel E. Horton, Francesca E. Duncan, Elnur Babayev

**Affiliations:** 1 Department of Obstetrics and Gynecology, Feinberg School of Medicine, Northwestern University, Chicago, Illinois, USA; 2 Galter Health Sciences Library & Learning Center, Feinberg School of Medicine, Northwestern University, Chicago, Illinois, USA; 3 Department of Earth, Environmental, and Planetary Sciences, Northwestern University, Evanston, Illinois, USA; 4 Department of Obstetrics and Gynecology, Northwestern Medicine Center for Fertility and Reproductive Medicine, Chicago, Illinois, USA

**Keywords:** increased temperature, reproduction, fertility, ovarian reserve, gamete, egg, granulosa cells

## Abstract

Climate change is driving significant environmental changes with profound implications for human health, including fertility. While the detrimental effects of heat on spermatogenesis are well-documented, the impact of elevated temperatures on ovaries and female fertility remains less explored. This review systematically examines the literature on heat stress (HS) effects on mammalian ovaries, follicles, and oocytes. Evidence from mammalian models indicates that HS significantly impairs ovarian function, disrupting hormone profiles, reducing ovarian size and weight, altering histology, decreasing granulosa cell viability, and compromising oocyte quality. Efforts to develop strategies and substances to mitigate these adverse effects are ongoing, but further research into the underlying mechanisms is urgently needed.

## Summary Sentence

This systematic review summarizes the evidence on the adverse effects of heat stress on the mammalian oocyte and ovary

## Introduction

The complex phenomena of climate change and global warming are the result of increasing greenhouse gas concentrations that have substantially altered the Earth’s environment and human vulnerability [1, 2]. Rising temperatures have resulted in altered weather patterns, more frequent and intense heatwaves, droughts, and wildfires, and in some locales worsening air quality, all leading to a global health crisis [3]. According to the World Health Organization (WHO), between 2030 and 2050, approximately 250,000 additional deaths per year will be due to climate change [4]. A report from the Red Cross found that in 2020, there were 132 unique extreme weather events with 51.6 million people having been affected globally and over 3,000 killed [5]. Overall, climate change has had and will continue to have profound implications on human health and well-being, with multifaceted impacts due to the varying geographical regions and socio-economic backgrounds of individuals. Additionally, vulnerable populations have been found to be even more susceptible to the effects of climate change [6]. This includes women, the elderly, children, pregnant women, individuals with pre-existing health conditions, and disinvested communities with limited access to resources.

Of the vulnerable populations, women and pregnant women are of particular interest as the impact of global warming and the exposure to high temperatures on fertility and reproduction is an area of increasing concern. Adverse effects of heat on spermatogenesis are well-recognized, including reduced sperm output, increased percentage of abnormal/aged sperm, reduced sperm counts and motility, and subfertility [7-9]. However, relatively less is known about the impact of elevated temperatures on female fertility and reproduction. Females are born with a finite number of oocytes, formed during fetal development, which comprise the ovarian reserve and dictates the reproductive lifespan [10]. At ~20 weeks of gestation, the fetal ovary contains a peak of millions of primordial follicles, each consisting of an immature oocyte surrounded by supportive somatic cells [11]. As females age, the total number of follicles declines and ends at menopause with the depletion of the ovarian reserve and cessation of reproductive capacity [12, 13]. Menopause is associated with increased risk of all-cause mortality, cardiovascular disease, metabolic syndrome, musculoskeletal diseases, and neurological dysfunction [14, 15]. Delayed childbearing and reproductive aging are also associated with the increase in infertility and miscarriage rates due to decline in gamete quality and quantity [16, 17]. Any environmental process that affects the ovarian reserve, rate of its decline, and quality of gametes could have profound effects on female reproduction and overall health. Increasing average global temperatures [18] and the frequency of days with extreme temperatures [19] may negatively affect female reproduction and reproductive aging. In this manuscript, we aimed to systematically compile and closely examine the literature on the impacts of elevated temperatures on the ovary and oocyte, and review potential mechanisms of and mitigating strategies for these effects.

## Materials and Methods

**Figure 1.**
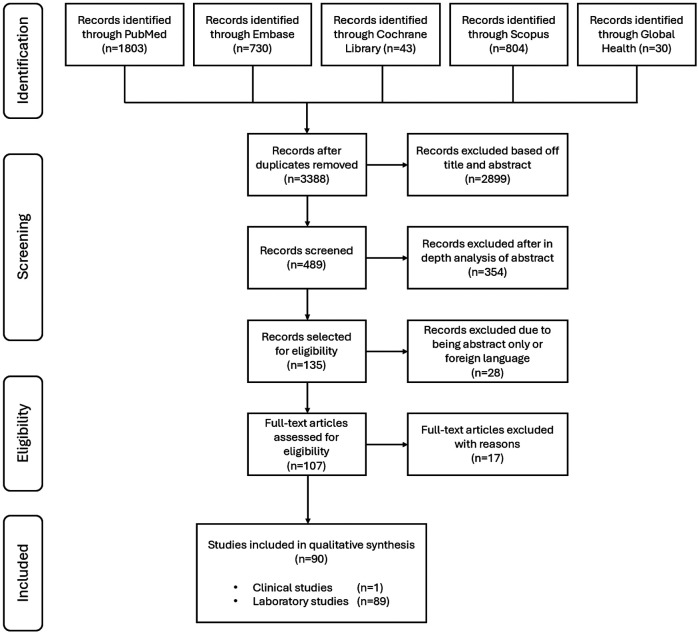
PRISMA flow chart of study selection. Ninety articles met all the inclusion criteria and were included in a qualitative synthesis. PRISMA, preferred reporting items for systematic review and meta-analysis. Seventeen studies were excluded after full text review as they either studied cooling effects, did not specify their HS paradigm, only focused on HS, or did not report controls, control temperatures, and/or the duration of the experimental intervention.

We performed a systematic review, and articles were identified through a search of PubMed (NIH/NLM), Embase (Elsevier), Cochrane Database of Systematic Reviews and Central Register of Controlled Trials (Wiley), Scopus (Elsevier), and Global Health (EBSCOhost) through October 17, 2024. The search strategy was designed by academic librarians (MB and KM) and involved an extensive list of key words and database-specific controlled vocabulary related to fertility, ovaries and heat stress (Supplementary Table S1). Eligible studies were limited to the English language, mammalian species, and full-text articles. Meeting abstracts, comments, case reports, news, and letters were excluded. There was no limitation on the date of publication and all citations through October 2024 were included. The review strategy followed the preferred reporting items for systematic review and meta-analysis (PRISMA) guidelines and is summarized in Fig. 1.

Out of the 3410 initially identified articles, 90 addressed the review scope through cross referencing using Rayyan [20] (LTZ and DG) and were fully extracted in the tabular form. Of the 90 included studies, 89 were laboratory studies and 1 was a clinical study (Fig. 1). This review only included the studies that investigated the effects of increased temperature on the ovary, follicle, oocyte, and/or granulosa cells, where both control and experimental groups were analyzed. Seventeen studies were excluded after full text review as they either studied cooling effects, did not specify their heat stress (HS) paradigm, or did not report controls, control temperatures, and/or the duration of the experimental intervention [21-37].

## Results

### The effects of heat stress on the ovary

Ovaries are responsible for generating female gametes and producing hormones regulating reproductive functions [38]. All studies investigating the effects of increased temperature on the ovary were conducted *in vivo*, (n=9) (summarized in Table 1) in a range of species from mouse to cow. Temperatures tested ranged from 18°C – 33.3°C for the control and 31°C – 43°C for the HS groups. Exposure time to HS ranged from 30 minutes per day to upwards of 35 days. One group conducted measurements comparing shade vs. sun exposure [39].

Chronic HS in mice results in smaller ovaries with partially contracted central portion and reduced numbers of blood vessels, when animals are exposed to a daily 1.5-h session of 39°C at 50% humidity for durations of 1, 3, or 6 weeks, compared to controls kept at a constant temperature of 25°C with 50% humidity for the same durations [40]. In sheep, HS also reduces ovarian weight, with estimates of mean ovarian weight being 1344 mg in control compared to 1159 mg in the hot-room exposed animals [41]. Here, sheep were exposed to HS of 40.6°C for 4h/day starting on day 1 that was gradually increased to 41.1°C for 8h/day by day 9, compared to controls that were consistently exposed to 32.2 – 33.3°C. The study investigating the effects of shade compared to sun exposure assigned cows and heifers at 160-190 days of gestation to shade (n=7 cows; n=2 heifers) or no shade (n=8 cows; n=2 heifers), followed by examination of the reproductive tract 7 days postpartum [39]. Interestingly, in these cows, ovarian volume is reduced when ovaries are adjacent to the uterine horn that contained a fetus, and HS by sun exposure prepartum attenuates this effect [39]. HS in rabbits causes histopathological changes in the ovary, including focal areas of fibroblast proliferation in the interstitial tissue and the presence of large open spaces [42]. At the protein level, HS leads to proteins that are altered in the ovaries of control and HS pigs [43, 44], and they were noted to include those involved with chemical detoxification, metabolism, and inflammation [43]. At a molecular level, HS increases ovarian mRNA abundance of insulin receptor (IRS1), protein kinase B subunit 1 (AKT1), low-density lipoprotein receptor (LDLR), luteinizing hormone receptor (LHCGR), and aromatase (CYP19a) after 7 days exposure of gilts to 35°C compared to constant thermoneutral temperature of 20°C [45]. These results demonstrate that HS may alter ovarian insulin-mediated PI3K signaling, which plays a role in follicle activation and viability [45]. Additionally, HS in pigs induces autophagy in the ovary, marked by the upregulation of autophagy-related proteins including BECN1 and LC3-II, which are involved in the degradation of dysfunctional intracellular machinery [46]. Finally, corpora lutea (CLs) sizes in pigs are significantly reduced in the HS group compared to the controls [47]. Overall, these data indicate that HS leads to the reduction of ovarian size and weight, alters its histological architecture, and may change the expression of genes involved in autophagy and follicle function.

### The effects of heat stress on reproductive hormone levels

Studies investigating the effects of increased temperature on hormone levels in mammals were conducted *in vivo* (n=8), *in vitro* (n=4), and both *in vivo & in vitro* (n=2) (summarized in Table 2). Temperatures tested ranged from 15°C – 38.5°C for the control and 28°C – 43°C for the HS groups. Exposure time to HS ranged from 2h per day to upwards of 28 days. One group studied the retrospective analysis of meteorological data from 2003-2007 [48].

Some studies in a bovine model report that progesterone (P4) levels are not affected or are lower [49-51], while other studies in cows, pigs, and sheep find that P4 secretion is higher after HS exposure compared to controls [47, 48, 52]. Most studies demonstrate decreased estradiol (E2) levels in HS compared to control animals (mice, rats, rabbits, pigs, goats, and cows [42, 49, 50, 52-56]), with only one study in cows reporting an increase [57]. HS in cows also results in higher serum follicle stimulating hormone (FSH) levels and decreased inhibin concentrations during the heat exposed estrous cycle [58]. Additionally, HS decreases aromatase expression in granulosa cells in mice, rats, and goats [53-55]. Notably, HS in goats leads to a significant delay in the luteinizing hormone (LH) surge [54]. In addition, HS leads to increased levels of follistatin (FST), which may reduce FSH and LH levels, indirectly affecting the late growth of dominant follicles [59]; in rabbits, FSH levels decrease in response to HS [42]. Overall, these findings suggest that HS induces alterations in reproductive hormone levels. Most studies report a decrease in aromatase expression and E2 levels, with conflicting data on P4 and FSH. Further studies are warranted to examine whether the changes in ovarian hormone levels are due to direct effects of HS on the ovaries or secondary to the alterations in pituitary hormone secretion or both.

### The effects of heat stress on the follicles

Follicles represent the functional units of the ovary. They produce a mature oocyte and gonadal hormones contributing to the regulation of the ovulatory cycle [11]. Studies investigating the effects of increased temperature on the follicles were conducted *in vivo* (n=11), *in vitro* (n=3), and both *in vivo & in vitro* (n=2) (summarized in Table 3). Temperatures tested ranged from 15°C – 38.5°C for control and 28°C – 42°C for the HS groups. Exposure time to HS ranged from 2h to 30 days. Some groups performed seasonal measurements (i.e., winter vs summer collections), direct solar radiation vs. shade, or retrospectively examined ambient temperatures [39, 48, 58, 60-64].

Interestingly, in cows, preovulatory follicles are cooler (0.74 ± 0.88°C) than neighboring uterine tissue and deep rectal temperatures under HS conditions suggesting a potential follicle-inherent thermoregulation mechanism [62]. HS in cows compromises folliculogenesis, with overall reduction in the size and number of follicles, fewer growing follicles, larger cohort of medium-sized follicles, and a decrease in the number of dominant follicles [58, 59, 61]. Preantral follicles, particularly those at the secondary stage (100.9 ± 13.7μm) of development, are the most sensitive to HS in cows, followed by early secondary (83.5 ± 8.4μm) and primary follicles (68.7 ± 9.2μm), indicating a stage-specific vulnerability to the increased temperatures [65]. Mechanistically, one study suggests that alterations of the activin-inhibin-follistatin system may partially be responsible for the tendency of compromised dominant follicle growth following HS [59].

Seasonal variations, perhaps at least partially mediated by temperature changes, influence the number of follicles in pigs. This results in the highest follicle numbers in mid-summer and the lowest in early fall [63]. In sheep, the diameter of the largest follicle is significantly higher during the breeding season (July) compared to winter (February) [48], which is in contrast to the dominant follicle diameter being smaller in summer compared to winter in cows [60]. Numerous studies in cows and goats also report a delayed deleterious effect of HS exposure on ovarian follicular growth [54, 58, 65]. In these models, HS reduces the viability and diameter of preantral follicles [54, 65]. In humans, a study in 631 subjects reports that higher ambient temperatures are linked to a lower antral follicle count (AFC), which is a measure of ovarian reserve. Specifically, a 1°C increase in average maximum temperature during the 90 days prior to ovarian reserve testing is associated with a 1.6% lower AFC. Additionally, this negative association is stronger November through June than during the summer months, suggesting the impact of possible acclimatization [64].

Evidence at histological and molecular level demonstrates that HS in mice leads to follicle atresia and dysregulated follicular development [40, 53], with oocytes detaching from the granulosa cell layer of antral follicles and granulosa cells becoming disordered and karyopyknotic [40]. Interestingly, HS in cows causes follicle cells to luteinize prematurely, which has been associated with persistent dominant follicles in cattle and accompanied by premature meiotic maturation of the oocyte, compromised subsequent embryo development, and fertility [52]. HS in pigs increases anti-apoptotic signaling in antral follicles [46]. Pig ovarian follicles constitutively express various heat shock proteins (HSPs) and heat shock factors (HSFs) in response to HS [66]. HSPs function to maintain cellular proteostasis and buffer against heat stressors [67]. HSFs are the transcription factors that regulate the expression of heat shock proteins [68]. In summary, folliculogenesis is significantly impaired in response to HS with most studies reporting a decrease in follicle numbers, size, and viability.

### The effects of heat stress on the oocyte

The oocyte is one of the largest cells in the body, is unique and extremely specialized, and undergoes dynamic processes during its maturation and development [69]. Most studies investigating the effects of increased temperature on the oocyte were conducted *in vitro* (n=38 *in vitro*, n=10 *in vivo*, n=5 *in vivo & in vitro*) (summarized in Table 4). From the 5 studies that examined HS *in vivo* and *in vitro*, 1 study looked at specific temperatures/duration *in vivo*, while the remaining 4 studies examined cold/winter vs. warm/summer months *in vivo*. Short term *in vivo* or *in vitro* exposure to HS (12h per day exposure to 32°C *in vivo* and 26h of maturation at 40°C *in vitro*) results in reduced porcine oocyte quality mediated in part by altered protein composition of the oocyte plasma membrane [70]. In cows, HS in summer months leads to impaired meiotic maturation, and compromised embryo cleavage, and blastocyst development rates [57, 71-73].

For the *in vivo* only studies, temperatures tested ranged from 19.5°C – 24°C for the control and 31°C – 36°C for the HS groups. Exposure time to HS ranged from 12.5h per day to 5 days. Most *in vivo* studies focus on seasonal temperature effects (n=7 out of 9). Seasonal HS in mice and cows leads to atypical oocyte morphologies, disrupted meiotic maturation, increase in the number of degenerated oocytes, and decrease in oocyte quality [61, 74, 75]. Seasonal HS in cows also leads to lower fertilization, embryo cleavage, and blastocyst development rates [76-78], with one study reporting that after HS exposure there is an increase in oocyte quality [79]. Results are conflicting in regard to HS impact on oocyte yield and *in vitro* maturation (IVM) in cows. Higher number of oocytes were obtained during the spring compared to summer in one study [75], whereas another study found collection numbers to be the highest during the summer [80]. After IVM, the percentage of mature, metaphase II (MII) eggs was not significantly different between seasons in one study [80]; however, another study found reduced oocyte maturation in the summer [77]. Lastly, HS in mice and pigs results in increased vacuolization in the oocyte, BCL21 protein abundance in the ovary, and decreased fetal size/development rate [46, 74]. Similar to the follicles, oocytes subjected to HS also exhibit delayed deleterious effects on gamete quality [78]. Embryo cleavage rates are decreased after *in vitro* fertilization (IVF) in cows [71]. At a molecular level, oocytes demonstrate significant changes in HSP and HSF expression levels in response to seasonal temperature variations [66]. In cow oocytes, when differentially expressed genes (DEGs) between germinal vesicle (GV) and MII stage oocytes were analyzed in May and July, only the July DEGs were enriched in “cellular responses to stress” pathway [81]. HS also increases anti-apoptotic signaling in pig oocytes [46].

For the *in vitro* studies, temperatures tested ranged from 37°C – 39°C for control groups and 38.5°C – 43°C for the HS groups. Exposure time to HS ranged from 15 minutes to 12 days. In cows, oocyte yield is decreased in HS compared control groups [82]. Additionally, oocyte-cumulus-granulosa complexes of cows exposed to HS (38.5-40.5°C) have significantly smaller mean oocyte diameters after IVM compared to the control group, indicating impaired oocyte growth due to heat exposure [83]. In a mouse study examining a range of elevated temperatures, oocyte maturation rates did not differ when exposed to 37-40°C. However, cytoplasmic maturation is impaired at 40°C, and nuclear formation is adversely affected beginning at 40.7°C [84]. Several studies in pig, cow, and buffalo did demonstrate decreased oocyte maturation with HS [85-94]. Similar to the *in vivo* studies, HS increases chromosome and spindle abnormalities in pig and cow oocytes *in vitro* [95, 96]. Three studies found that HS leads to the reduced oocyte mitochondrial membrane potential (MMP) [94, 97, 98]. Numerous studies report the decrease in embryo cleavage and blastocyst formation rates as a result of HS exposure in mice, pigs, and cows [82-84, 86, 89, 90, 93, 94, 98-113]. Interestingly, severe HS in cow oocytes at 43°C for up to 60 minutes during IVM followed by IVF showed that while blastocyst formation rates are not affected by HS for up to 30 minutes, longer exposures (45 and 60 minutes) significantly reduces both blastocyst and expanded blastocyst formation rates [104]. This indicates a threshold beyond which heat shock begins to negatively affect oocyte viability and developmental potential. Interestingly, cow blastocyst development rates improve when IVF is performed earlier in heat stressed eggs [114]. At a cellular and molecular level, cumulus-oocyte-complex (COC) HS exposure during IVM doesn’t change RNA abundance in oocytes, surrounding cumulus cells, or in 4- to 8- cell embryos but increases total RNA in resultant blastocysts after IVF [115]. Similar to the *in vivo* studies, several studies in pigs and cows demonstrate that HS during IVM induces apoptotic events in oocytes, as well as increases ROS and oxidative stress [91, 92, 97-99, 105, 106, 113, 116-118], with one study identifying members of the Bcl-2 family of peptides as having a protective effect [98]. Additionally, a possible breed-specific resilience to HS was reported. Compared to Jersey cows, HS in Holstein cows appears to adversely affect nuclear maturation at a higher degree, results in increased ROS levels, and abnormal oocyte mitochondria distribution suggesting impaired cytoplasmic maturation [118]. HS in cow oocytes at 41-41.5°C results in a significant increase in TUNEL-positive cells [97, 105, 116]. Antioxidant gene expression analysis in heat-stressed cow oocytes reveals that the expression GPX1, MnSOD, and G6PD tends to be upregulated [112, 119]. Overall, the evidence is overwhelming across species that HS detrimentally affects oocyte meiotic maturation, quality, and developmental competence both *in vivo* and *in vitro*.

### Effects of heat stress on the granulosa cells

Granulosa cells (GCs) support oocyte growth and development, ensure ovulation of developmentally competent gamete, and secrete hormones that regulate multiple organismal functions [120]. Studies that examined the impact of heat stress on GCs were conducted both *in vivo* and *in vitro* studies (n=4 *in vivo*, n=4 *in vitro*, n=1 *in vivo & in vitro*) (summarized in Table 5). For the *in vivo* studies, temperatures tested ranged from 15°C – 25°C for the control and 28°C – 42°C for the HS groups. Exposure time to HS ranged from 3h per day to 4 days. For the *in vitro* studies, temperatures ranged from 37°C – 38°C for the control and 39°C – 43°C for the HS groups. Exposure time to HS ranged from 2h to 24h.

HS in cows and buffalos reduces the viability of GCs compared to controls [50, 121]. HS in pigs increases the incidence of vacuolization in GCs [46]. HS exposure in mice and cows results in increased granulosa cell apoptosis [49, 50, 53, 122]. However, one study reported that HS in cows does not induce apoptosis in GCs but affects the expression of genes encoding the extracellular matrix proteins, glycoproteins, proteins playing a role in lipid synthesis, metabolic processes, and apoptosis [59]. HS in rats inhibits FSH-R expression in GCs, and this is associated with enhanced susceptibility to apoptosis [55]. At a molecular level, somatic components of the periovulatory follicle (i.e., cumulus cells surrounding the maturing oocyte and the mural GCs lining the follicle wall) demonstrate altered gene expression in response to varying degrees of elevated body temperatures, with majority of gene ontologies related to protein processing, the endoplasmic reticulum or Golgi apparatus [123]. Cow GCs cultured under HS (40.5°C) demonstrate altered gene expression with upregulated genes (*BCL-2, BAX, HSP*) involved in apoptosis regulation, and downregulated genes (*SF-1*, *CYP19A1, STAR, CYP11A1)* involved in secretion of E2 and P4 [122]. To summarize, HS in GCs reduces cell viability, increases apoptosis, and dysregulates gene expression.

### Interventions to mitigate the adverse effects of heat stress on granulosa cells, oocytes, and embryos

Studies investigating the effects of supplements to mitigate the sequelae of heat stress on mammalian GCs, oocytes, and embryos were conducted *in vivo* (n=7) and *in vitro* (n=15) (summarized in Table 6). Administration of Moringa oleifera aqueous seed extracts during HS in rabbits increases E2 and FSH [42]. Injection of pregnant mare serum gonadotropin (PMSG) during HS in rats results in decreased aromatase, E2, and FSH-R of GCs [55]. The addition of rapamycin during oocyte IVM in pigs under HS results in increased oocyte maturation rates [88]. Supplementation of FSH during IVM under HS in cows, improves oocyte maturation rates and embryo development [124]. Supplementation with astaxanthin during HS in pigs increases oocyte maturation, fertilization, and blastocyst development [86]. Addition of 10% follicular fluid to the oocyte maturation medium in cows, during IVM under HS, results in increased blastocyst development rates [108]. Treatment with Selenium during cow COC IVM under HS improves oocyte maturation and embryo cleavage rates, and the total number of blastocysts, as well as upregulates the expression of antioxidant genes (*CCND1, SEPP1, GPX-4, SOD, CAT*) [125]. Idebenone (IDB), potent antioxidant that functions as a carrier in electron transport chain, protects pig oocytes from HS during IVM (20-24 h, 42°C) through improving cumulus cell expansion, nuclear maturation, and blastocyst development rates [126]. Treatment with cysteine in COCs during IVM in cows under HS results in increased GSH levels and blastocyst development rates [83]. Addition of IGF-1 during IVM under HS in cows results in improved maturation, embryo cleavage rates, MMP, and decreases apoptotic cells [97, 105]. Treatment with anti-apoptotic peptides, BH4 domain of Bcl-xL (TAT-BH4), Bax inhibitor peptide (BIP), or combination of the two (BIP-BH4) decreases apoptosis and improves blastocyst development of heat stressed cow oocytes [98]. In cows, the addition of z-DEVD-fmk (group II caspase inhibitor) and sphingosine 1-phosphate blocks the negative effects of HS on embryo cleavage and subsequent development [91, 109, 110]. Supplementation of melatonin during HS in pigs increases total follicle numbers [63]. Supplementation with linoleic and linolenic acids significantly decreases ROS levels during pig oocyte IVM under HS conditions (40-42h under HS of 41.5°C) [117]. Additionally, in cows, treatment of GCs with a standardized extract of asparagus officinalis stem improves mitochondrial activity, reduces oxidative stress and increases the formation of lipid droplets, which are important for cholesterol storage used in steroid hormone production [51]. In pigs, while serum prolactin (PRL) did not differ from controls to HS only treated, when HS is supplemented with the addition of zearalenone (ZEN), there is significant increase in PRL [43]. In the same study, E2 was initially decreased with HS; however, when HS is supplemented with ZEN, there was a reduction in decrease of E2 [43]. In pigs, treatment of heat stressed COCs with Mogroside V attenuates deleterious effects of HS on oocyte maturation and blastocyst formation [127]. Additionally, Vitamin C supplementation to culture media in HS cow granulosa cells results in decreased ROS and apoptosis, and increased P4 and E2 [128]. Overall, investigators are seeking strategies to improve the detrimental impact of HS on mammalian oocytes and embryos. Although some compounds showed promise, further investigations and understanding of their mechanisms of actions are needed.

## Conclusions

**Figure 2.**
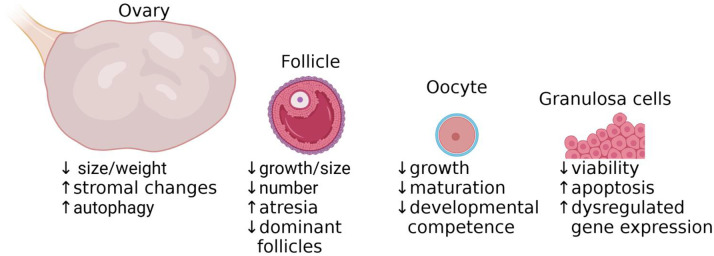
Schematic summarizing the evidence on heat stress effects on the ovary, follicle, oocyte, and granulosa cells.

There are significant adverse impacts of HS on the ovaries, reproductive hormone profiles, follicles, oocytes, and GCs in mammals (Figure 2). HS results in dysregulated gene expression and increases apoptosis in multiple ovarian cell types. In the ovary, HS reduces ovarian size and weight, disrupts histology, and induces autophagy. In the follicles, HS leads to the increased follicle atresia, reduced size and number of follicles, and a decrease in the number of dominant follicles. In the oocytes, HS negatively impacts oocyte growth, maturation, and developmental competence. In the GCs, HS results in significantly decreased viability and increased apoptosis. These findings underscore the multifaceted impact of HS on ovarian function. With the projected increase in high-temperature days in the coming decades, elucidating the mechanisms by which HS affects the ovary is critical. Current knowledge of the molecular pathways involved remains limited, necessitating further research to inform the development of effective therapeutic interventions. Efforts to identify compounds that mitigate HS-induced damage to oocytes and embryos are ongoing, highlighting the urgency of advancing strategies to preserve reproductive health under thermal stress.

## Supplementary Material

Supplement 1

## Figures and Tables

**Table 1.** Effects of heat stress on the ovary

Study	Species	Heat Exposure Methods	Main Findings
Ryle M, 1963	Sheep	In vivo; wet (32.2-33.3°C) vs. dry (40.6-41.1°C), 4-8h/d, 26d	↓ ovarian size
Lewis GS, et al., 1984	Cow	In vivo; shade vs. no shade, 30d	↓ ovarian volume near previously gravid uterine horn, HS attenuates the decrease in ovarian volume
Nteeba J, et al., 2015	Pig	In vivo; 20°C vs. 35°C, 7d or 35d	↑ *IRS1*, AK1, LDLR, LHCGR, and CYP19A1
Hale BJ, et al., 2017	Pig	In vivo; 19.5-20.5°C vs. 31°C, 5d	↑ BECN1, LC3B-II; ↓ autophagy related complex
Bidne KL, et al., 2019	Pig	In vivo; 20 ± 1 °C vs. 31.6-35 ± 1 °C, 12h/d, 10d	↓ CL size
Bei M, et al., 2020	Mouse	In vivo; 25°C vs. 39°C, 1.5h/d, 1,3,6 wk; 43°C, 0.5h, 1,3,6, or 12h	↓ ovarian growth; ↑ HSP
Mutwedu VB, et al., 2022	Rabbit	In vivo; 18-24°C vs. 35-36°C, 8h/d for 2 wk	↑ ovarian histopathology (large open spaces), fibroblast proliferation
Roach CM, et al., 2024	Pig	In vivo; 35.0±0.2°C (12h) and 32.2±0.1°C (12h) vs. 21.0±0.10°C, 7d	↑ ovarian proteins involved in chemical detoxification, metabolism, and inflammation
Roach CM, et al., 2024	Pig	In vivo; 35.0±0.2°C (12h) and 32.2±0.1°C (12h) vs. 21.0±0.10°C, 7d	Alters ovarian proteome

Abbreviations: Insulin receptor substrate 1 (IRS1), adenylate kinase 1 (AK1), low-density lipoprotein receptor (LDLR), luteinizing hormone/chorionic gonadotropin receptor (LHCGR), cytochrome P450 Family 19 Subfamily A Member 1 (CYP19A1), beclin-1 (BECN1), microtubule associated protein light chain 3 beta II (LC3B-II), corpora lutea (CL), heat shock protein (HSP),

**Table 2.** Effects of heat stress on reproductive hormones

Study	Species	Heat Exposure Methods	Main Findings
Sammad A, et al., 2022	Cow	In vitro; 38°C vs. 43°C, 2h	↓ E2, P4
Bridges PJ, et al., 2005	Cow	In vitro; 37°C vs. 39°C, 41°C, 96h	↓ androstenedione, E2; ↑ P4
Khan A, et al., 2020	Cow	In vitro; 38°C vs. 39-41 °C, 2h	↓ E2, P4
Ho KT, et al., 2023	Cow	In vitro; 38.5°C vs. 41°C, 12h	~ P4
Bidne KL, et al., 2019	Pig	In vivo; 20 ± 1 °C vs. 31.6-35 ± 1 °C, 12h/d, 10d	↑ P4 to CL weight
Ozawa M, et al., 2005	Goat	In vivo; 25°C vs. 36°C, 48h	↓ *Cyp19A1*, E2; delayed LH surge
Roth Z, et al., 2000	Cow	In vivo; cooled vs. direct solar radiation, 7h/d	↑ FSH, ↓ inhibin
Hashem NM, et al., 2011	Sheep	In vivo; meteorological data 2003-2007	↑ P4
Vanselow J, et al., 2016	Cow	In vivo; 15°C vs. 28°C, 4d	↓ E2
Shimizu T, et al., 2005	Rat	In vivo; 25°C vs. 35°C, 4d	↓ *Cyp19A1*, E2, FSH-R
Mutwedu VB, et al., 2022	Rabbit	In vivo; 18-24°C vs. 35-36°C, 8h/d, 2 wk	↓ E2, FSH
Li J, et al., 2016	Mouse	In vivo; 25°C vs. 42°C, 3h/d for 7-28d, In vitro; 37°C vs. 41-42°C for 2-24h	↓ *Cyp19A1*, E2
Maya-Soriano MJ, et al., 2013	Cow	In vivo; cold vs. warm months In vitro; 38°C vs. 41.5°C, 3h	↑ E2
Roach CM, et al., 2024	Pig	In vivo; 35.0±0.2°C (12h) and 32.2±0.1°C (12h) vs. 21.0±0.10°C, 7d	↓ E2

Abbreviations: estradiol (E2), progesterone (P4), corpora lutea (CL), cytochrome P450 Family 19 Subfamily A Member 1 (Cyp19A1), luteinizing hormone (LH), follicle stimulating hormone (FSH), follicle stimulating hormone receptor (FSH-R), Prolactin (PRL)

**Table 3.** Effects of heat stress on follicles

Study	Species	Heat Exposure Methods	Main Findings
Roth Z, et al., 2000	Cow	In vivo; cooled vs. direct solar radiation, 7h/d	↑ medium-sized (6-9mm) follicles
Ozawa M, et al., 2005	Goat	In vivo; 25°C vs. 36°C, 48h	↓ follicular size; no ovulations
Shehab-El-Deen MA, et al., 2010	Cow	In vivo; winter vs. summer	↓ dominant follicle diameter
Hashem NM, et al., 2011	Sheep	In vivo; meteorological data 2003-2007	↑ diameter of the largest follicle
Alves BG, et al., 2014	Cow	In vivo; winter vs. summer	↓ follicles
Vanselow J, et al., 2016	Cow	In vivo; 15°C vs. 28°C, 4d	↓ diameters of dominant follicles
Lopez-Gatius F, & Hunter R, 2017	Cow	In vivo; shade vs. direct sun radiation, 12d	↓ mean follicle diameter; no ovulations
Arend LS, et al., 2019	Pig	In vivo; mid summer, late summer, vs. early fall	Gilts: ↑ follicle in mid summer, ↓ in early fall; Sows: ↑ in late summer
Gaskins AJ, et al., 2021	Human	In vivo; ambient temperatures measured year round	↓ AFC
Bei M, et al., 2020	Mouse	In vivo; 25°C vs. 39°C, 1.5h/d, 1,3,6 wk; 43°C, 0.5h, 1,3,6, or 12h	oocytes detaching, GC disordered and karyopyknotic
Hale BJ, et al., 2017	Pig	In vivo; 19.5-20.5°C vs. 31°C, 5d	↑ anti-apoptotic signaling
Bridges PJ, et al., 2005	Cow	In vitro; 37°C vs. 39-41°C, 96h	premature luteinization
Aguiar LH, et al., 2020	Cow	In vitro; 38.5°C vs. 41°C, 8h/d, 7d	↓ viability and diameter of preantral follicles, ↑ *SOD1* and *BAX*
Kawano K, et al., 2022	Cow	In vitro: 38.5°C vs. HS (incremental increase 38.5-40.5°C for 5-9h each), 12d	HS during early antral stage associated with ↓fertility & oocyte competence
Li J, et al., 2016	Mouse	In vivo; 25°C vs. 42°C, 3h/d In vitro; 37°C vs. 42°C, 24h, or 37°C vs. 41°C, 2h	↑ atretic follicles, ↑ apoptosis
Pennarossa G, et al., 2012	Pig	In vivo; summer vs. winter In vitro; 38.5°C vs. 41.5°C, 1h	↑ HSP and HSF

Abbreviations: antral follicle count (AFC), granulosa cell (GC), superoxide dismutase (SOD1), BCL2-associated X protein (BAX), heat stress (HS), heat shock protein (HSP), heat shock factor (HSF)

**Table 4.** Effects of heat stress on the oocyte

Study	Species	Heat Exposure Methods	Main Findings
Berger T & Roberts BM, 2009	Pig	In vivo; 20-22°C vs. 32°C, 12h/d, 7d In vitro; 38.5°C vs. 40°C, 26-44h	In vivo; ↓ POMP In vitro; ↓ POMP, fertilization rate
Maya-Soriano MJ, et al., 2013	Cow	In vivo; cold vs. warm months In vitro; 38°C vs. 41.5°C, 3h	In vivo; ↑ anomalous maturation In vitro; ↑ anomalous MII morphology, anomalous CG distribution pattern
Pavani K, et al., 2015	Cow	In vivo; cold vs. warm months In vitro; 38.5°C vs. 39.5-40.5°C, 24h	In vivo; ↓ meiotic maturation, cleavage rate, embryonic development In vitro; ↓ meiotic maturation, nuclear maturation, cleavage rate, embryonic development
Pavani KC, et al., 2016	Cow	In vivo; cold vs. warm months In vitro; 38.5°C vs. 39.5-40.5°C, 6-24h	In vivo; ↓ meiotic maturation, cleavage rate, embryonic development, *DNMT1, HSPA14, Cx43* In vitro; ↓ nuclear maturation rate, meiotic maturation, *HSPA14, CDH1*; ↑ *DNMT1*
Pavani KC, et al., 2017	Cow	In vivo; winter vs. summer In vitro; 38.5°C vs. 39.5°C, 6-24h	In vivo; ↓ *HSPA14* In vitro; ↑ *CDH1, DNMT1*
Baumgartner AP & Chrisman CL, 1987	Mouse	In vivo; 21±3°C vs. 35±1°C, 12.5h	↑ atypical oocyte morphology and maturation, pre-implantation loss, embryo mortality; ↓ fetal size, fetal development rate
Al-Katanani YM, et al., 2002	Cow	In vivo; winter vs. summer (+ cool vs. minimal shade), 42d	↑ cleavage rate; ↓ blastocyst development
Gendelman M & Roth Z, 2012	Cow	In vivo; cold vs. hot seasons	↓ fertilization rate, cleavage rate, blastocyst development, MII egg *MOS, GDF9*, *POU5F1*, *GAPDH*
Alves BG, et al., 2014	Cow	In vivo; winter vs. summer	↑ degenerated and morphologically damaged oocytes
Hale BJ, et al., 2017	Pig	In vivo; 19.5-20.5°C vs. 31°C, 5d	↑ vacuolization, BCL2L1
Kanwichai S, et al., 2019	Cow	In vivo; cool vs. summer vs. rainy seasons	↑ COCs and oocytes
Diaz FA, et al., 2021	Cow	In vivo; spring vs. summer	↓ high quality oocytes; altered gene expression with RNA-Seq
Báez F, et al., 2022	Cow	In vivo; winter vs. summer	↓ GV3 rate, blastocyst yield/quality; ↑ apoptotic index
Ozmen O & Karaman K, 2023	Cow	In vivo; spring vs. summer, GV vs. MII oocyte RNA-Seq	DEGs enrichment in “cellular responses to stress” pathway in July
Maddahi A, et al., 2024	Cow	In vivo; summer vs. winter	↑ oocyte quality
Edwards JL & Hansen PJ, 1996	Cow	In vitro; 39°C vs. 41-42°C, 12h or 24h	↓ cleavage rate, blastocyst development, protein synthesis; ↑ HSP68 synthesis
Edwards JL & Hansen PJ, 1997	Cow	In vitro; 39°C vs. 41°C, 12h	↓ oocytes, blastocyst development, protein synthesis, HSP70
Ju JC, et al., 1999	Cow	In vitro; 39°C vs. 40.5-43°C, 15-60m	↓ blastocyst formation and hatching
Roth Z & Hansen PJ, 2004	Cow	In vitro; 38.5°C vs. 40-41°C, 12h	↓ cleavage rate, blastocyst development; ↑ DNA fragmentation, caspase activity
Roth Z & Hansen PJ, 2004	Cow	In vitro; 38.5°C vs. 41°C, 12h	↓ cleavage rate, blastocyst development
Ju JC & Tseng JK, 2004	Pig	In vitro; 39°C vs. 41.5°C, 1-4h	↑ chromosome and spindle abnormalities, fragmented chromatin
Tseng JK, et al., 2004	Cow	In vitro; 38.5°C vs. 41.5°C, 1-4h	↑ chromosome and spindle abnormalities
Payton RR, et al., 2004	Cow	In vitro; 38.5°C vs. 41°C, 6-24h	↑ type III cortical granules; ↓ MII rate, blastocyst development
Roth Z & Hansen PJ, 2005	Cow	In vitro; 38.5°C vs. 40-41°C, 12h	↓ MII rate; ↑ apoptotic oocytes
Schrock GE, et al., 2007	Cow	In vitro; 38.5°C vs. 41.0°C, 12h	↑ blastocyst development when IVF performed earlier
Wang JZ, et al., 2009	Mouse	In vitro; 37°C vs. 38.5-41°C, 14h	↓ nuclear formation, cytoplasmic maturation, blastocyst formation
Edwards JL, et al., 2009	Cow	In vitro; 38.5 °C vs. 41°C, 12h	↓ blastocyst development
Soto P & Smith LC, 2009	Cow	In vitro; 39°C vs. 41°C, 21h	↓ embryo development; ↑ apoptosis; loss of oocyte MMP
Kalo D & Roth Z, 2011	Cow	In vitro; 38.5°C vs. 41°C, 22h	↓ MII rate, cleavage rate, blastocyst formation; ↑ annexin-V binding
Payton RR, et al., 2011	Cow	In vitro; 38.5°C vs. 41°C, 12h	↑ total RNA in blastocysts
Pennarossa G, et al., 2012	Pig	In vivo; summer vs. winter In vitro; 38.5°C vs. 41.5°C, 1h	↑ HSP and HSF
Balboula AZ, et al., 2013	Cow	In vitro; 38.5°C vs. 41°C, 17h	↑ cathepsin B, caspase 3 cathepsin B, caspase 3, apoptosis
Do LT, et al., 2015	Pig	In vitro; 38.5°C vs. 41°C, 46h	↓ maturation, blastocyst development
Meiyu Q, et al., 2015	Cow	In vitro; 38.5°C vs. 41.5°C, 22h	↑ apoptosis; ↓ resumption of meiosis, cleavage rate
Ascari IJ, et al., 2017	Cow	In vitro; 38.5°C vs. 41°C, 12h	↑ apoptosis, ROS production; ↓ MMP, oocyte developmental competence
El-Sayed A, et al., 2018	Buffalo	In vitro; 38.5°C vs. 39.5-40.5°C, 6h	↓ maturation rate, cleavage rate, blastocyst development; ↓ *HSF2, HSP70, BAX, p53, SOD1, COX1, MAPK14*; ↑ *HSP90, HSF1*
Payton RR, et al., 2018	Cow	In vitro; 38.5°C vs. 41°C, 12h	↓ blastocyst development, cytoplasmic ROS; ↑ mitochondrial ROS, ATP; Δ in relative abundance of 52 transcripts
Rodrigues TA, et al., 2019	Cow	In vitro; 38.5°C vs. 41°C, 14h	↓ cumulus cell expansion, cleavage rate, blastocyst development
Báez F, et al., 2019	Cow	In vitro; 38.5°C vs. 41°C, 6-22h	↓ MII rate, fertilization rate, blastocyst yield
Cavallari F, et al., 2019	Cow	In vitro; 38.5°C vs. 41°C, 3h or 22h	↓ cleavage rate
Camargo LSA, et al., 2019	Cow	In vitro; 38.5°C vs. 41°C, 12h	↓ PN formation, cleavage rate
Amaral CS, et al., 2020	Cow	In vitro; 38.5°C vs. 40.5°C, 22-24h	↓ cleavage rates, blastocyst rates, IFNT; ↑ ROS
Pöhland R, et al., 2020	Cow	In vitro; 38.5°C vs. 40°C, 24h	↓ cleavage rates, blastocyst formation, HSP90; ↑ HSP70 in cumulus cells
Stamperna K, et al., 2020	Cow	In vitro; 39°C vs. 41°C, 6h	↓ cleavage rate, embryo formation; altered mRNA abundance and gene expression
Yaacobi-Artzi S, et al., 2020	Cow	In vitro; 38.5°C vs. 41.5°C, 22h	↑ ROS; ↓ meiotic resumption, MII rate
Hale BJ, et al., 2021	Pig	In vitro; 38.5°C vs. 41°C, 21h	↓ maturation rate; ↑ autophagy
de Souza VdGP, et al., 2021	Cow	In vitro; 38.8°C vs. 41°C, 12h	↓ nuclear maturation, cleavage, embryo development, *DROSHA*; ↑ bta-miR-19b abundance
Stamperna K, et al., 2021	Cow	In vitro; 39°C vs. 41°C, 6h	↓ cleavage rates, blastocyst rates; ↑ HSP90AA1
Kawano K, et al.,2022	Cow	In vitro; 38.5°C vs. HS (incremental increase 38.5-40.5°C for 5-9h each), 12d	↓ oocyte diameters, blastocyst development, GSH
Current JZ, et al., 2022	Pig	In vitro; 38.5°C vs. 41.5°C, 40-42h	↑ ROS
Lee J, et al., 2023	Cow	In vitro; 38.5°C vs. 40.5°C, 21h	↓ nuclear maturation; ↑ ROS, peripheral distribution of mitochondria
Wrzecińska M, et al., 2023	Cow	In vitro; 38.5°C vs. 38.3-41°C, 6-12h	↓ cleavage rate, blastocyst development, LC3 and SIRT1; ↑ cytoplasmic and mitochondrial ROS
Mzedawee HRH, et al., 2024	Cow	In vitro; 38.5°C vs. 39.5-40.5°C, 23h	↓ MII rate, cleavage rates, blastocyst development
Feng X, et al., 2024	Cow	In vitro; 38.5°C vs. 41.0°C, 12h	↓ MII rate, cleavage rates, blastocyst development, MMP; ↑ ROS, apoptosis

Abbreviations: porcine oocyte membrane protein (POMP), metaphase of meiosis II (MII), cortical granule (CG), DNA methyltransferase 1 (DNMT1), Heat Shock Protein Family A [Hsp70] Member 14 (HSPA14), connexin 43 (Cx43), MOS proto-oncogene, serine/threonine kinase (MOS), growth differentiation factor-9 (GDF9), POU domain, class 5, transcription factor 1 (POU5F1), glyceraldehyde-3-phosphate dehydrogenase (GAPDH), BCL2-like 1 (BCL2L1), cumulus-oocyte-complexes (COCs), RNA sequencing (RNA-Seq), germinal vesicle stage 3 where chromatin condenses into a single clump within the nuclear envelope (GV3), heat shock protein 68 (HSP68), heat shock protein (HSP70), in vitro fertilization (IVF), mitochondrial membrane potential (MMP), heat shock protein (HSP), heat shock factor (HSF), reactive oxygen species (ROS), heat shock factor 2 (HSF2), BCL2-associated X protein (BAX), tumor protein p53 (p53), superoxide dismutase 1 (SOD1), cyclooxygenase 1 (COX1), mitogen-activated protein kinase 14 (MAPK14), heat shock protein 90 (HSP90), heat shock factor 1 (HSF1), pronuclei (PN), interferon-tau (IFNT), Drosha Ribonuclease III (DROSHA), heat shock protein 90 alpha family class A member 1 (HSP90AA1), glutathione (GSH), microtubule-associated protein 1A/1B-light chain 3 (LC3), Sirtuin 1 (SIRT1)

**Table 5.** Effects of heat stress on granulosa cells

Study	Species	Heat Exposure Methods	Main Findings
Li J, et al., 2016	Mouse	In vivo; 25°C vs. 42°C 3h/d In vitro: 37°C vs. 42°C, 24h or 37°C vs. 41°C, 2h	↑ apoptotic GCs; Caspase-3, Bim
Shimizu T, et al., 2005	Rat	In vivo; 25°C vs. 35°C, 4d	↑ apoptosis; ↑ *Bax*
Vanselow J, et al., 2016	Cow	In vivo; 15°C vs. 28°C, 4d	↑ *HAS3, CRHBP*
Hale BJ, et al., 2017	Pig	In vivo; 19.5-20.5°C vs. 31°C, 5d	↑ BCL2L1, vacuolization
Klabnik JL, et al., 2022	Cow	In vivo; temperature humidity index (THI) of 65.8±0.1 vs. 71.0-86.4, 12.1±0.2h	87 DEGs; DEGs suggested to promote ovulation, impact collagen formation or angiogenesis
Li L, et al., 2016	Cow	In vitro; 38°C vs. 40.5°C, 2h	1211 DEGs; ↑ apoptosis through BAX/BCL-2 pathway; ↓ steroidogenic gene mRNA expression
Khan A, et al., 2020	Cow	In vitro; 38°C vs. 39°C, 40°C, 41°C, 2h	142 DEGs (39°C); 321 DEGs (40°C); 294 DEGs (41°C); ↓ viability; ↑ apoptosis, ROS
Faheem MS, et al., 2021	Buffalo	In vitro; 37°C vs. 39.5°C, 40.5°C, 41.5°C, 24h	↓ viability, miR-1246, miR-181a, miR27b; ↑ SOD2, miR-708
Sammad A, et al., 2022	Cow	In vitro; 38°C vs. 43°C, 2h	12,385 DEGs; ↑ ROS, apoptosis, stress-response genes

Abbreviations: granulosa cells (GCs), B-cell Lymphoma 2-like protein 11 (Bim), BCL2-associated X protein (Bax), Hyaluronan Synthase 3 (HAS3), Corticotropin Releasing Hormone Binding Protein (CRHBP), BCL2-like 1 (BCL2L1), differentially expressed genes (DEGs), B-cell leukemia/lymphoma 2 (BCL-2), reactive oxygen species (ROS), superoxide dismutase 2 (SOD2)

**Table 6.** Interventions to mitigate heat stress impacts on ovarian function

Study	Species	Heat Exposure Methods + Intervention	Main Findings
Mutwedu VB, et al., 2022	Rabbit	In vivo; 18-24°C vs. 35-36°C, 8h/d, 2wk + Moringa oleifera aqueous seed extract	↑ E2 & FSH
Ho KT, et al., 2023	Cow	In vitro; 38.5°C vs. 41°C, 12h + EAS	↑ P4; ↑ STAR, 3β-HSD, mitochondrial activity, lipid droplet formation; ↓ ROS
Shimizu T, et al., 2005	Rat	In vivo; 25°C vs. 35°C, 4d + PMSG	↓ aromatase activity, E2, FSH-R
Arend LS, et al.,2019	Pig	In vivo; mid summer (26.2°C), late summer (28.3°C), vs. early fall (27.4°C) + melatonin	↑ follicles
Maddahi A, et al., 2024	Cow	In vivo; summer vs. winter + vitamins E, C, and coenzyme Q10	↑ maturation, cleavage rates
Roth Z & Hansen PJ, 2004	Cow	In vitro; 38.5°C vs. 40- 41°C, 12h + z-DEVD-fmk	blocks HS effect on embryo cleavage rate and blastocyst development
Roth Z & Hansen PJ, 2004	Cow	In vitro; 38.5°C vs. 41°C, 12h + S1P	blocks HS effect on cleavage and subsequent development
Roth Z & Hansen PJ, 2005	Cow	In vitro; 38.5°C vs. 40- 41°C, 12h	S1P blocks the effect of HS on progression through meiosis
Soto P & Smith LC, 2009	Cow	In vitro; 39°C vs. 41°C, 21h + BIP, TAT-BH4, or BIP+BH4	↑ HS oocytes development to blastocysts; ↓ embryonic development, ↓ apoptosis
Do LT, et al., 2015	Pig	In vitro; 38.5°C vs. 41°C, 46h + astaxanthin	↑ oocyte maturation, fertilization, blastocyst development; ↓ proportion of apoptotic cells in oocytes exposed to oxidative stress
Meiyu Q, et al., 2015	Cow	In vitro; 38.5°C vs. 41.5°C, 22h + IGF-1	↑ proportion of MII-stage eggs and 4-cell-stage cleaved embryos
Ascari IJ, et al., 2017	Cow	In vitro; 38.5°C vs. 41°C, 12h + IGF-1	↑ MMP, ROS production; ↓ apoptotic cells
Rodrigues TA, et al., 2019	Cow	In vitro; 38.5°C vs. 41°C, 14h + 10% follicular fluid	↑ blastocyst development
Karaşahi n T, 2020	Cow	In vivo; autumn vs. spring + FSH	↑ maturation & embryonic growth rates
Hale BJ, et al., 2021	Pig	In vitro; 38.5°C vs. 41°C, 21h + rapamycin	↑ the maturation rate
Shi XY, et al., 2022	Pig	In vitro; 38.5°C vs. 42°C, 4h + IDB	↑ quality and developmental competence of oocytes, antioxidant capacity, GDF9 and BMP15; protects mitochondrial function
Kawano K, et al., 2022	Cow	In vitro; 38.5°C control vs HS (incremental increase 38.5-40.5°C for 5-9h each), 12d + cysteine	↑ GSH levels, blastocyst rate
Current JZ, et al., 2022	Pig	In vitro; 38.5°C vs. 41.5°C, 40-42h + linoleic and linolenic acids	↓ ROS; ↑ fertilization rates, cleavage rates, blastocyst formation rates, antioxidative enzymes (SOD and catalase), GSH
Toosinia S, et al., 2023	Cow	In vitro; 38.5°C vs. 41°C HS, 24h + Se	↑ viability of cumulus cells and oocytes, MII rate, cleavage rate, blastocyst development, antioxidative defense genes; ↓ apoptosis-related genes
Roach CM, et al., 2024	Pig	In vivo; 35.0±0.2°C (12h) and 32.2±0.1°C (12h) vs. 21.0±0.10°C, 7d + ZEN	↑ Prolactin, ↑E2
Peng K, et al., 2024	Pig	In vitro; 38.5°C vs. 42°C + MV	↑ MII rate, blastocyst rate
Sammad A, et al., 2024	Cow	In vitro; 38°C vs. 43°C, 2h + Vitamin C	↓ ROS, apoptosis; ↑ P4, E2

Abbreviations: Estradiol (E2), follicle stimulating hormone (FSH), Extract of Asparagus officinalis stem (EAS), progesterone (P4), steroidogenic acute regulatory protein (STAR), 3 beta-hydroxysteroid dehydrogenase (3β-HSD), reactive oxygen species (ROS), pregnant mare serum gonadotropin (PMSG), follicle stimulating hormone receptor (FSH-R), caspase-3 inhibitor (z-DEVD-fmk), heat stress (HS), sphingosine 1-phosphate (S1P), anti-apoptotic peptides BH4 domain of Bcl-xL (TAT-BH4), Bax inhibitor peptide (BIP), insulin-like growth factor 1 (IGF-1), metaphase of meiosis II (MII), mitochondrial membrane potential (MMP), idebenone (IDB), glutathione (GSH), selenium (Se), zearalenone (ZEN), Mogroside V (MV)
